# Retrospective study on the association between carotid plaque stability assessed by ultrasound imaging features and stroke risk

**DOI:** 10.3389/fneur.2025.1638989

**Published:** 2026-01-22

**Authors:** Bo Wang, Yingjun Li, LiSha Qiu

**Affiliations:** 1 Department of Ultrasound Medicine, Xiamen Fifth Hospital, Xiamen, Fujian, China; 2 Department of Joint Surgery, Xiamen Fifth Hospital, Xiamen, Fujian, China; 3 Department of Administration and Personnel, Xiamen Fifth Hospital, Xiamen, Fujian, China

**Keywords:** carotid plaque, elastography, neovascularization, plaque stability, stroke risk, ultrasound imaging

## Abstract

**Background:**

Carotid plaque instability is a critical determinant of ischemic stroke risk. Identifying ultrasound imaging features associated with plaque vulnerability may enhance stroke risk stratification.

**Methods:**

This retrospective cohort study evaluated 225 patients (≥18 years) with carotid plaques identified by Doppler ultrasound at a tertiary hospital between January 2022 and December 2024. Patients were followed for 1 year and grouped by stroke outcomes. Baseline demographics, laboratory data, medication use, plaque characteristics (echogenicity, anatomical location, length, thickness, surface morphology, calcification, and intraplaque hemorrhage), intima-media thickness (IMT), neovascularization (graded by contrast-enhanced ultrasound), and plaque hardness (quantified by elastography) were analyzed.

**Results:**

Stroke patients exhibited plaques with medium to high echogenicity, location at the carotid bifurcation, length >10 mm, intraplaque hemorrhage, moderate/severe calcification, higher neovascularization grades, and lower proportions of hard regions (all *p* < 0.05). Multivariate regression identified medium to high echogenicity, bifurcation location, plaque length >10 mm, intraplaque hemorrhage, higher neovascularization grade, and lower plaque hardness as independent predictors of stroke risk.

**Conclusion:**

Ultrasound imaging features of carotid plaques (echogenicity, location, length, intraplaque hemorrhage, neovascularization grade, and plaque hardness) are independently associated with stroke risk. These findings support the integration of advanced ultrasound imaging into clinical assessments to improve ischemic stroke risk stratification.

## 1 Introduction

Stroke remains a leading cause of morbidity, mortality, and long-term disability worldwide, with ischemic stroke accounting for the majority of cases (1). A substantial proportion of ischemic strokes are caused by extracranial carotid artery atherosclerosis, underscoring the importance of carotid artery pathology in cerebrovascular events (2). Early identification of patients at heightened stroke risk due to carotid atherosclerosis is therefore critical for implementing effective prevention strategies (3).

Traditional risk stratification for carotid atherosclerosis has primarily relied on assessing luminal stenosis severity using ultrasonography or angiographic methods, as established by landmark trials such as the North American Symptomatic Carotid Endarterectomy Trial (NASCET) and the European Carotid Surgery Trial (ECST) (4). However, emerging evidence highlights that plaque vulnerability, rather than stenosis alone, plays a pivotal role in ischemic stroke pathogenesis (5). Vulnerable plaques are characterized by specific histopathological features, including large lipid cores, a thin fibrous cap, intraplaque hemorrhage, neovascularization, and inflammation, all of which increase the risk of rupture and thromboembolism (6). Nevertheless, different studies highlight that plaque pathology, in addition to stenosis, is essential to understand in the pathogenesis of ischemic stroke. Up to 30% of ischemic strokes occur in patients with high-risk non-stenotic plaques characterized by thin soft caps, ruptured or ulcerated inflamed fibrous caps, hemorrhagic intraplaque hemorrhage (IPH), active inflammatory processes, and large lipid-rich necrotic cores. IPH is plaque strongest predictor of ischemic event and poor prognosis, regardless of the degree of stenosis (7–9).

Carotid ultrasonography has emerged as a sophisticated diagnostic modality, enabling *in vivo* assessment of plaque composition and stability (10). Advanced techniques, including contrast-enhanced ultrasound (CEUS) and elastography, facilitate detailed evaluations of plaque echogenicity, neovascularization, and mechanical properties, thereby enhancing risk prediction capabilities (11). Nonetheless, the prognostic significance of these imaging features is still under investigation. Historically, luminal stenosis was regarded as the primary predictor of stroke risk; however, accumulating evidence suggests that ultrasound-derived plaque characteristics (such as echolucency, surface ulceration, intraplaque hemorrhage, and neovascularization) offer prognostic insights independent of stenosis severity.

In a prospective study, Huang et al. (2024) identified a distinctive CEUS perfusion pattern where microbubbles penetrated from the superficial plaque surface into the plaque interior, correlating with a stroke risk of 2.32 (95% CI: 1.175, 4.594) (12). A meta-analysis of CEUS studies further indicated that plaque enhancement was significantly greater in stroke patients compared to controls (13). Different studies have demonstrated concordance between ultrasound features and histologically verified intraplaque hemorrhage, characterized by hypoechoic plaque cores, irregular plaque surfaces, and heightened neovascularization (14–16). In addition, retrospective cohort data suggest that plaques exhibiting intraplaque hemorrhage and ulceration are associated with markedly increased risk, even in cases of moderate stenosis (17). These findings indicate the value of ultrasound-based plaque characterization in enhancing risk stratification beyond mere reliance on stenosis severity.

Despite these advancements, a substantial gap persists in the clinical application of comprehensive plaque characterization for risk assessment in asymptomatic carotid artery disease (18). Alternative imaging modalities, such as carotid MRI and computed tomography angiography (CTA), may provide additional structural and compositional insights; however, their routine clinical application remains limited (19, 20). Recent studies indicate that while MRI provide detailed compositional analysis and CTA facilitates luminal assessment, factors such as cost, restricted availability, and the necessity for contrast agents hinder their integration into standard screening protocols, particularly in primary care settings (21, 22). In contrast, ultrasound is a non-invasive and widely accessible method capable of evaluating various plaque characteristics, including echogenicity, intraplaque hemorrhage, and mechanical attributes pertinent to plaque stability and rupture vulnerability. Emerging ultrasound-derived biomarkers, such as intraplaque neovascularization, gray-scale median (GSM), and plaque stiffness, have been established as independent predictors of cerebrovascular outcomes. Multimodal ultrasound approaches, which combine superb microvascular imaging with shear-wave elastography, have revealed that extensive intraplaque neovascularization and reduced plaque stiffness are associated with an increased risk of poor functional outcomes 90 days post-ischemic stroke or transient ischemic attack (TIA). CEUS provides a precise evaluation of intraplaque neovascularization, correlating well with microvessel density, and is augmented by GSM quantification, which characterizes plaque tissue and enhances reproducibility in cerebrovascular risk stratification (23–25).

This study aims to address this gap by analyzing the prognostic value of ultrasound-detected plaque phenotypes in relation to stroke risk. The integration of these imaging biomarkers into clinical practice holds the potential to improve plaque and stroke risk management through enhanced risk stratification, earlier identification of at-risk individuals, and the implementation of targeted preventive interventions. In doing so, this study advances risk prediction models by emphasizing plaque phenotype rather than stenosis severity, a shift that may substantially improve precision care in the management of cerebrovascular disease.

## 2 Methods

### 2.1 Study design

We analyzed 225 patients with carotid plaques detected through ultrasound screening at the Xiamen Fifth Hospital (Xiamen, China) between January 2022 and December 2024. Over a one-year follow-up period, participants were stratified by stroke outcomes: 144 remained stroke-free compared to 81 who experienced cerebrovascular events.

Before conducting analyses, we removed all personal identifiers from patient records following hospital privacy protocols. Only clinically relevant information related to the study objectives was retained, ensuring that no individual patient could be identified. This study did not involve any intervention or alteration to standard medical care; all procedures and analyses were conducted retrospectively using existing records. Therefore, the study posed no additional risk to participants.

The Ethics Committee of the Xiamen Fifth Hospital approved the study (protocol: 0734-0022). Considering the retrospective design and the use of de-identified data, the committee granted an exemption from the requirement for written informed consent.

Ultrasound assessments were conducted by three highly trained sonographers: two senior radiologists with over 10 years of vascular ultrasound experience and one attending physician with 6 years of experience. To verify reproducibility, intra- and inter-observer kappa analyses were performed on 20% of scans, confirming high reproducibility with kappa values exceeding 0.82.

The diagnosis of stroke events followed the criteria of the American Heart Association and American Stroke Association, requiring acute focal neurological deficits lasting 24 h or more, confirmed by examination from a board-certified neurologist and supported by neuroimaging (CT and/or MRI). Transient ischemic attacks were excluded unless acute ischemic lesions were confirmed on imaging (26).

### 2.2 Inclusion and exclusion criteria

Inclusion criteria for this study encompassed patients aged 18 years or older, identified with at least one carotid plaque through color Doppler ultrasonography (27), exhibiting no significant psychiatric or cognitive impairments, and without major diseases that would significantly affect quality of life or life expectancy, such as advanced malignancy or end-stage cardiac disease. All patients were required to have complete medical records, including basic clinical information, imaging results, and laboratory data.

Exclusion criteria included a history of definitive cardioembolic sources, such as atrial fibrillation or valvular heart disease, or small perforator lesions causing stroke. Patients with poor-quality ultrasound images that prevented accurate characterization of plaques were excluded. Individuals with a history of neck radiotherapy or surgery that could alter plaque morphology were also excluded. Additional exclusion criteria included renal insufficiency, severe hepatic dysfunction, acute inflammatory or infectious diseases, pregnancy or breastfeeding, drug abuse, major surgery within the preceding 3 months, and incomplete one-year follow-up or missing follow-up data.

### 2.3 Data sources

#### 2.3.1 Baseline data

Demographic baseline information such as age and gender, along with patients’ medical and pharmaceutical histories, were retrieved from the electronic medical records created during clinic visits. The EMR data capture the patients’ medical status at the time of presentation. Other variables, including vascular risk factors, duration of comorbidities, and subsequent clinical assessments, were cross-verified for completeness and internal consistency within the outcome categories.

#### 2.3.2 Laboratory data

Blood tests were conducted using a fully automated biochemical analyzer, the URIT-8600 (Urit Medical Electronic Co., Ltd., China). The instrument automatically processed samples and generated quantitative outputs.

#### 2.3.3 Plaque morphological characteristics

High-frequency linear array probes (Philips EPIQ 7G, Philips Healthcare, Netherlands) were used to scan the carotid arteries and observe plaque echogenicity. Ultrasound imaging revealed distinct plaque characteristics: hypoechoic plaques appeared as darker areas, typically indicating lipid-rich cores or recent hemorrhage, whereas brighter medium-to-high echogenic regions suggested fibrous tissue or calcification. The evaluation protocol examined three features. First, surface contours were assessed to distinguish smooth surfaces from those with ulceration or irregular protrusions. Second, internal composition was analyzed, including calcification distribution. Third, calcification severity was classified: mild cases showed scattered fine echogenic foci, whereas moderate to severe cases demonstrated dense coarse calcifications associated with reduced structural stability.

We conducted plaque assessments using the GE Logiq E9 (GE Healthcare, USA) ultrasound system, focusing on two key dimensions: thickness and length. To determine maximum thickness, the digital caliper tool measured from the intima to adventitia at each plaque’s widest visible cross-section. For length measurements, we tracked the entire lesion from proximal to distal endpoints. Accuracy was ensured through multiplanar verification (taking longitudinal, transverse, and oblique views) with only the highest measurements retained. During scanning, operators systematically traced the carotid artery path to precisely document each plaque’s anatomical position.

To characterize altered hemodynamics, Color Doppler imaging (Philips EPIQ 7G) was used to evaluate plaque flow patterns and morphology, following established ultrasonography guidelines (28). Doppler ultrasound was chosen over standard B-mode imaging due to its ability to visualize intraplaque flow signals, detect ulcerations, and identify flow disturbances that are indicative of plaque instability. Unlike B-mode, which provides only structural information, Doppler imaging offers functional assessment, allowing for enhanced detection of microvascular activity and neovascularization within plaques. This functional insight improves risk stratification by identifying plaques more prone to rupture and cerebrovascular events.

#### 2.3.4 Intima-media thickness

Intima-media thickness (IMT) assessment used high-frequency linear array probes with longitudinal neck scanning. After identifying the common carotid artery (CCA) and bifurcation, a 1–2 cm arterial segment proximal to the bifurcation was selected for measurement. The electronic caliper captured dual-wall measurements (near and far walls) at this standardized site, with the averaged value representing the final IMT quantification.

#### 2.3.5 Neovascularization assessment

The protocol used high-frequency linear array probes for initial carotid artery mapping to identify plaque location and morphology. Upon plaque detection, intravenous contrast administration was immediately followed by real-time low mechanical index (MI < 0.2) ultrasound imaging to monitor intralesional enhancement patterns. Neovascularization was classified into four grades based on previous criteria (29, 30):

- Grade 0: No contrast signals (avascular plaque)
- Grade 1: Sparse punctate/linear signals (early angiogenesis)
- Grade 2: Multiple discrete signals forming focal reticular patterns (moderate vascularity)
- Grade 3: Confluent reticular networks with globular enhancement (advanced neovascularization)

These categories are widely used in CEUS-based carotid plaque evaluation.

#### 2.3.6 Plaque hardness

Operators first localized carotid plaques using high-frequency linear array probes, documenting their precise positions and structural features. Standardized plaque compression was then achieved through either dedicated manual devices or calibrated probe-applied pressure. Maintaining consistent pressure across measurements ensured uniform tissue deformation, which is essential for reliable comparisons. Elastography imaging subsequently generated real-time, color-coded tissue stiffness maps. Sequential image capture under varying pressure conditions followed predefined image-quality criteria, with distorted frames prompting immediate repeat acquisition to maintain analytical integrity. The software calculated Young’s modulus values based on changes in applied pressure and tissue deformation.

Imaging analysis software was then used to segment the elastographic images, separating the hard regions (blue areas) from the soft regions (red areas). The software automatically generated the percentage of hard regions relative to total plaque area.

### 2.4 Data analysis

For the statistical analysis, we used SPSS version 29.0 (IBM Corp., Armonk, NY, USA) to process and analyze the collected data. Categorical variables were summarized using frequencies and percentages [n (%)]. For assessing categorical data, we employed the chi-square test (χ^2^). Continuous data that followed a normal distribution were reported as means ± standard deviations (M ± SD). Between-group comparisons for normally distributed continuous data were conducted using independent samples t-tests. To assess the relationship between continuous variables, Pearson’s correlation analysis was performed. Multivariable logistic regression techniques were used to adjust for possible confounding (age, sex, history of hypertension, diabetes, lipid profile, smoking status). Variance inflation factors were evaluated to assess multicollinearity. Model performance was assessed using the area under the receiver operating characteristic curve (AUC) and the Hosmer–Lemeshow goodness-of-fit test. Key effect estimates were accompanied by 95% confidence intervals. A *p*-value < 0.05 was used to determine statistical significance.

## 3 Results

### 3.1 Basic information

Baseline characteristics were analyzed by comparing groups with and without stroke, as shown in Table 1. Overall, the groups were similar in age, sex, BMI, waist circumference, and other lifestyle factors and comorbidities. The prevalence of prior cerebrovascular events was higher in the stroke group (18.52% vs. 8.33%, χ^2^ = 5.132, *p* = 0.023), indicating that prior cerebrovascular disease may contribute to increased stroke risk.

**Table 1:** Baseline characteristics from the stroke group and the non-stroke group.

Index	Non-stroke group (n = 144)	Stroke group (n = 81)	t/χ^2^	*p*-value
Age (years)	57.74 ± 7.54	59.48 ± 8.25	1.604	0.110
BMI (kg/m^2^)	22.98 ± 1.26	23.21 ± 1.44	1.283	0.201
Waist Circumference (cm)	90.71 ± 4.36	91.66 ± 4.87	1.516	0.131
Sex			0.716	0.398
Female	58 (40.28%)	28 (34.57%)		
Male	86 (59.72%)	53 (65.43%)		
Educational attainment			0.251	0.882
Primary school or below	29 (20.14%)	18 (22.22%)		
Middle school	59 (40.97%)	34 (41.98%)		
University or above	56 (38.89%)	29 (35.80%)		
Alcohol consumption history	76 (52.78%)	51 (62.96%)	2.187	0.139
Smoking history	52 (36.11%)	36 (44.44%)	1.512	0.219
Physical activity	70 (48.61%)	36 (44.44%)	0.361	0.548
Atrial fibrillation	6 (4.17%)	4 (4.94%)	0	1.000
Hypertension	68 (47.22%)	48 (59.26%)	3.007	0.083
Diabetes mellitus	18 (12.50%)	15 (18.52%)	1.500	0.221
Coronary heart disease	28 (19.44%)	18 (22.22%)	0.246	0.620
Hyperlipidemia	65 (45.14%)	39 (48.15%)	0.189	0.664
Family history of cardiovascular disease	35 (24.31%)	27 (33.33%)	2.116	0.146
Family history of stroke	24 (16.67%)	22 (27.16%)	3.510	0.061
History of cerebrovascular events	12 (8.33%)	15 (18.52%)	5.081	0.024

### 3.2 Laboratory data

No significant differences were observed in lipid profiles, glycemic markers, renal function parameters, or systemic inflammatory markers between the groups, as shown in Table 2.

**Table 2:** Comparison of laboratory parameters between stroke and non-stroke groups.

Index	Non-stroke group (*n* = 144)	Stroke group (*n* = 81)	*t/χ* ^2^	*p*
Total cholesterol (mmol/L)	4.74 ± 0.96	4.89 ± 0.91	1.150	0.251
LDL-C	3.35 ± 0.68	3.51 ± 0.74	1.611	0.109
HDL-C	1.43 ± 0.42	1.33 ± 0.34	1.956	0.052
Triglycerides	1.34 ± 0.27	1.41 ± 0.36	1.499	0.136
Fasting blood glucose (mmol/L)	4.98 ± 0.39	5.06 ± 0.41	1.455	0.147
Serum creatinine (μmol/L)	83.57 ± 13.79	86.44 ± 12.62	1.545	0.124
Uric acid (μmol/L)	361.78 ± 60.83	366.68 ± 63.06	0.572	0.568
CRP (mg/L)	3.52 ± 0.69	3.69 ± 0.75	1.766	0.079

LDL-C: Low-Density Lipoprotein Cholesterol; HDL-C: High-Density Lipoprotein Cholesterol; CRP: C-Reactive Protein.

### 3.3 Medication use

**Figure 1:**
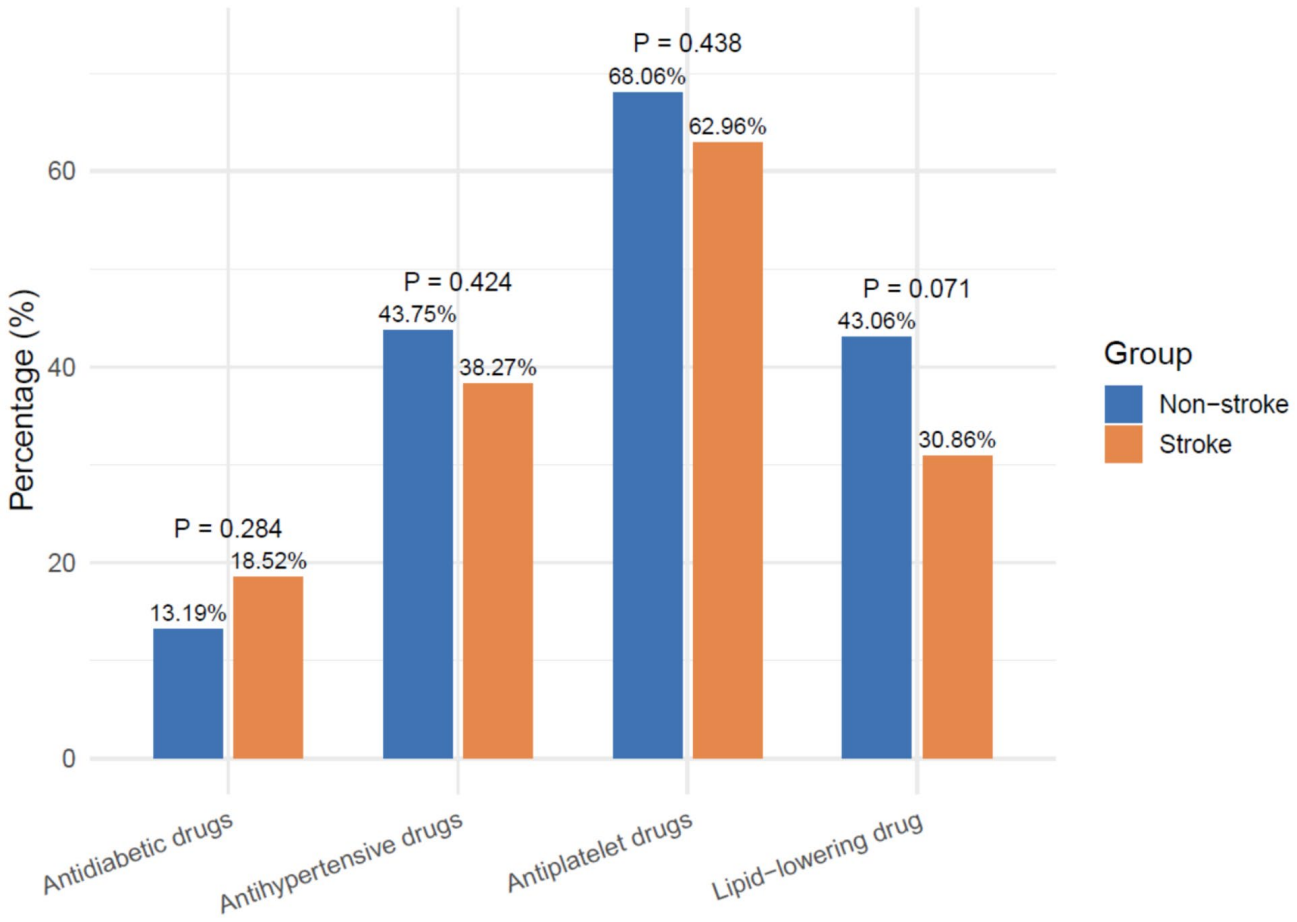
Medication use in stroke and non-stroke groups.

Medication use was comparable, as shown in Figure 1. Lipid-lowering drugs were taken by 30.86% of stroke patients versus 43.06% of non-stroke patients, and no significant differences were observed in antiplatelet, antihypertensive, or antidiabetic therapy.

### 3.4 Morphological characteristics of arterial plaques

Plaque features differed significantly, as shown in Table 3. Among stroke patients, 77.78% of plaques were of medium to high echogenicity, whereas 54.86% of plaques in the control group exhibited medium to high echogenicity (χ^2^ = 11.694, *p* < 0.001). In addition, plaques were located at the carotid bifurcation in 66.67% of stroke patients versus 47.92% of controls (χ^2^ = 7.354, *p* = 0.007). Furthermore, 41.98% of stroke patients had plaques longer than 10 mm, compared with 25.69% of controls (χ^2^ = 6.362, *p* = 0.012). Intraplaque hemorrhage was detected in 40.74% of stroke patients, whereas only 22.22% of controls exhibited this feature (χ^2^ = 8.654, *p* = 0.003). Lastly, the proportion of patients with moderate to severe calcification was higher in the stroke group (38.27%) (χ^2^ = 4.878, *p* = 0.027).

**Table 3:** Morphological characteristics of arterial plaques.

Index	Non-stroke group (*n* = 144)	Stroke group (*n* = 81)	*t/χ^2^*	*p*
Echogenicity distribution			11.694	<0.001
Low echogenicity	65 (45.14%)	18 (22.22%)		
Medium to high echogenicity	79 (54.86%)	63 (77.78%)		
Plaque thickness (mm)	2.06 ± 0.16	2.11 ± 0.23	1.548	0.124
Surface morphology distribution			3.802	0.051
Smooth	102 (70.83%)	47 (58.02%)		
Irregular or ulcerated	42 (29.17%)	34 (41.98%)		
Location distribution			7.354	0.007
Carotid bifurcation	69 (47.92%)	54 (66.67%)		
Other sites (carotid bulb or proximal internal carotid artery)	75 (52.08%)	27 (33.33%)		
Plaque length (mm)	9.36 ± 0.91	9.75 ± 0.85	3.116	0.002
Proportion of plaques with length greater than 10 mm	37 (25.69%)	34 (41.98%)	6.362	0.012
Intraplaque hemorrhage proportion	32 (22.22%)	33 (40.74%)	8.654	0.003
Calcification degree distribution			4.878	0.027
None or mild	109 (75.69%)	50 (61.73%)		
Moderate or severe	35 (24.31%)	31 (38.27%)		

### 3.5 Intima-media thickness

**Figure 2:**
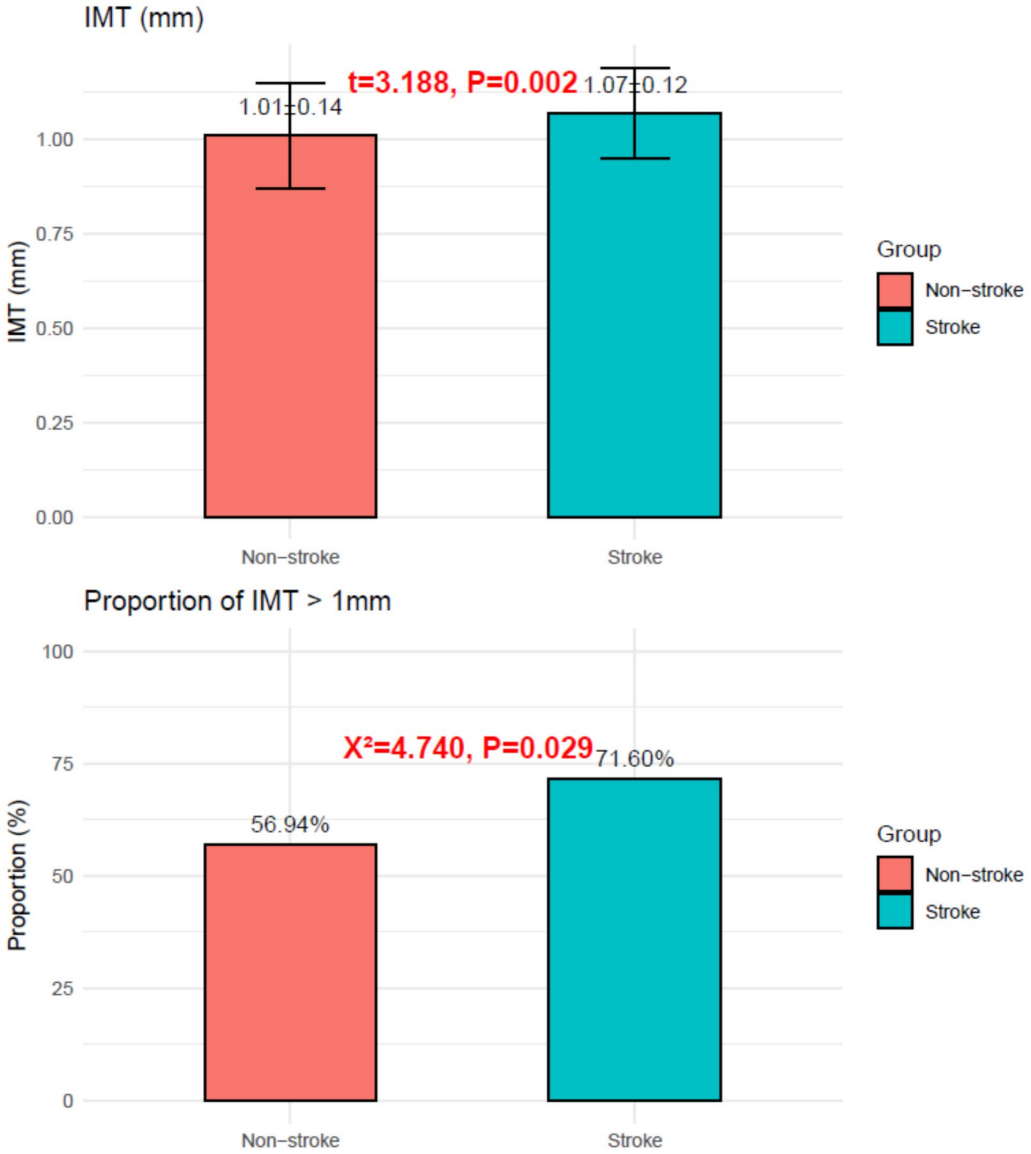
Intima-media thickness in the stroke group and the non-stroke group.

Stroke patients had higher IMT (1.07 ± 0.12 mm vs. 1.01 ± 0.14 mm; *p* = 0.002) and a greater proportion with IMT > 1 mm (71.60% vs. 56.94%; χ^2^ = 4.740, *p* = 0.029), as shown in Figure 2.

**Figure 3:**
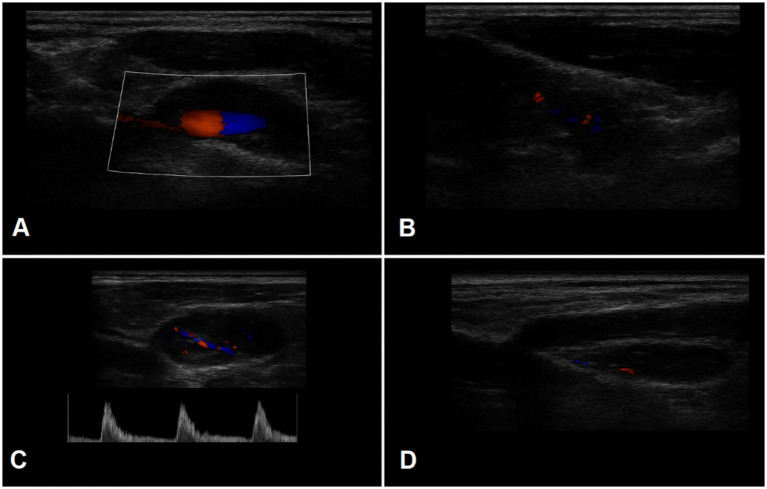
Representative Doppler ultrasound images of carotid plaques: **(A)** Color Doppler image showing a plaque with moderate echogenicity and smooth surface morphology. **(B)** High-grade intraplaque neovascularization visualized as multiple microvascular flow signals within the plaque core. **(C)** Plaque located at the carotid bifurcation, demonstrating disturbed flow patterns and elevated peak systolic velocity. **(D)** Long-axis Doppler view illustrating a plaque length >10 mm with areas of flow voids suggestive of intraplaque hemorrhage.

Representative Doppler ultrasound images illustrating the key plaque features assessed (echogenicity, neovascularization, bifurcation location, and plaque length) are shown in Figure 3. These representative images correspond to the plaque features analyzed in the multivariable model and highlight the added value of Doppler imaging in characterizing plaque vulnerability.

### 3.6 Neovascularization

**Figure 4:**
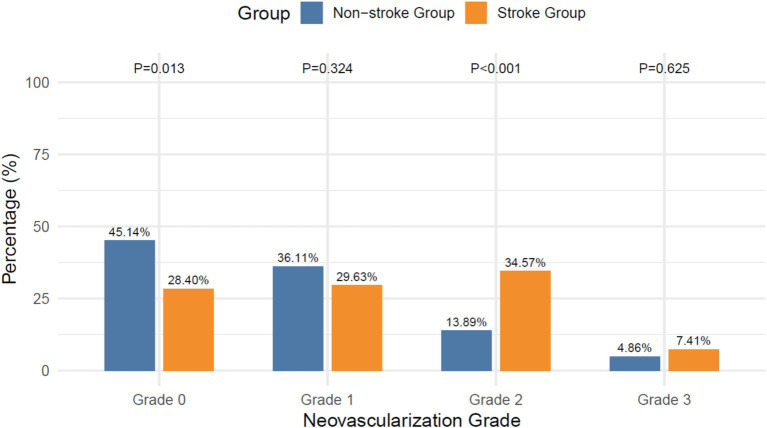
Intraplaque neovascularization grades.

The stroke group showed higher prevalence 2 neovascularization (34.57% vs. 13.89%; χ^2^ = 13.209, *p* < 0.001) and lower grade 0 prevalence (28.40% vs. 45.14%; χ^2^ = 6.103, *p* = 0.013), with no differences observed in grades 1 or 3, as shown in Figure 4.

### 3.7 Plaque hardness

**Figure 5:**
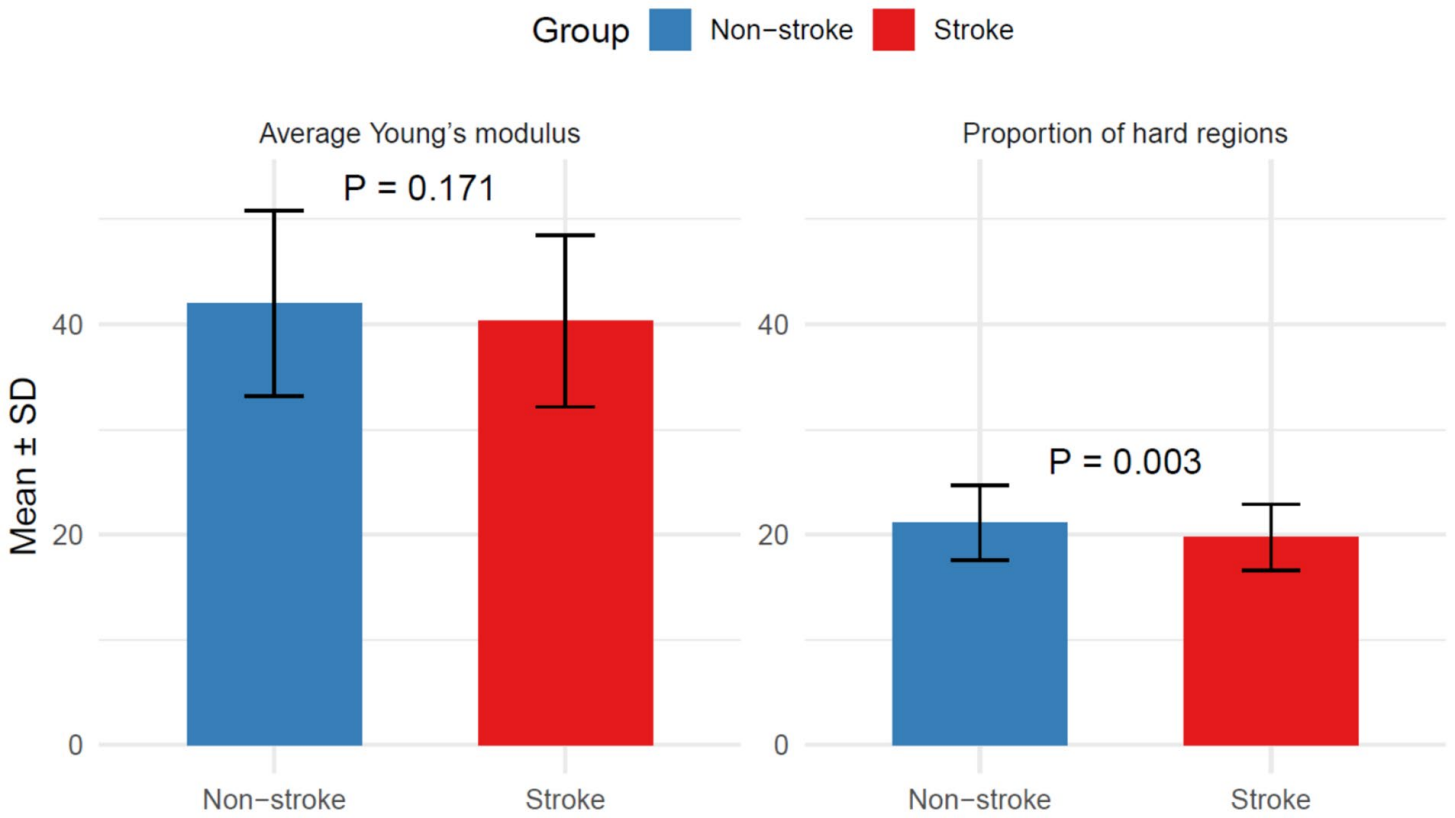
Plaque hardness in the stroke group and the non-stroke group.

Average Young’s modulus was similar (40.33 ± 8.17 vs. 42.01 ± 8.84; *p* = 0.171), but stroke patients had lower proportion of hard regions (19.75 ± 3.14 vs. 21.15 ± 3.55; *p* = 0.003), as shown in Figure 5.

### 3.8 Regression analysis

Univariate logistic regression showed that several ultrasound plaque features were significantly linked to stroke risk. These included plaque location, echogenicity, intraplaque hemorrhage, plaques ≥10 mm, severe calcification, IMT > 1 mm, higher grades of neovascularization, and a smaller proportion of hard regions as shown in Table 4.

**Table 4:** Logistic regression analysis of ultrasound plaque features associated with stroke risk.

Index	Coefficient	Std error	OR (95% CI)	*p*
Echogenicity distribution (Low/Medium-High)	−1.06	0.32	0.35 (0.18, 0.64)	<0.001
Location distribution (Carotid bifurcation/Other)	0.78	0.29	2.17 (1.24, 3.87)	0.01
Plaque Length >10 mm	0.74	0.30	2.09 (1.17, 3.74)	0.01
Intraplaque hemorrhage	0.88	0.30	2.41 (1.33, 4.37)	0.00
Calcification degree (None/Mild vs. Moderate/Severe)	−0.66	0.30	0.52 (0.29, 0.93)	0.03
IMT > 1 mm	0.65	0.30	1.91 (1.07, 3.47)	0.03
Neovascularization Grade (0/1 vs. 2/3)	1.14	0.31	3.14 (1.71, 5.80)	<0.001
Proportion of hard regions	−0.12	0.04	0.89 (0.82, 0.96)	0.00

**Figure 6:**
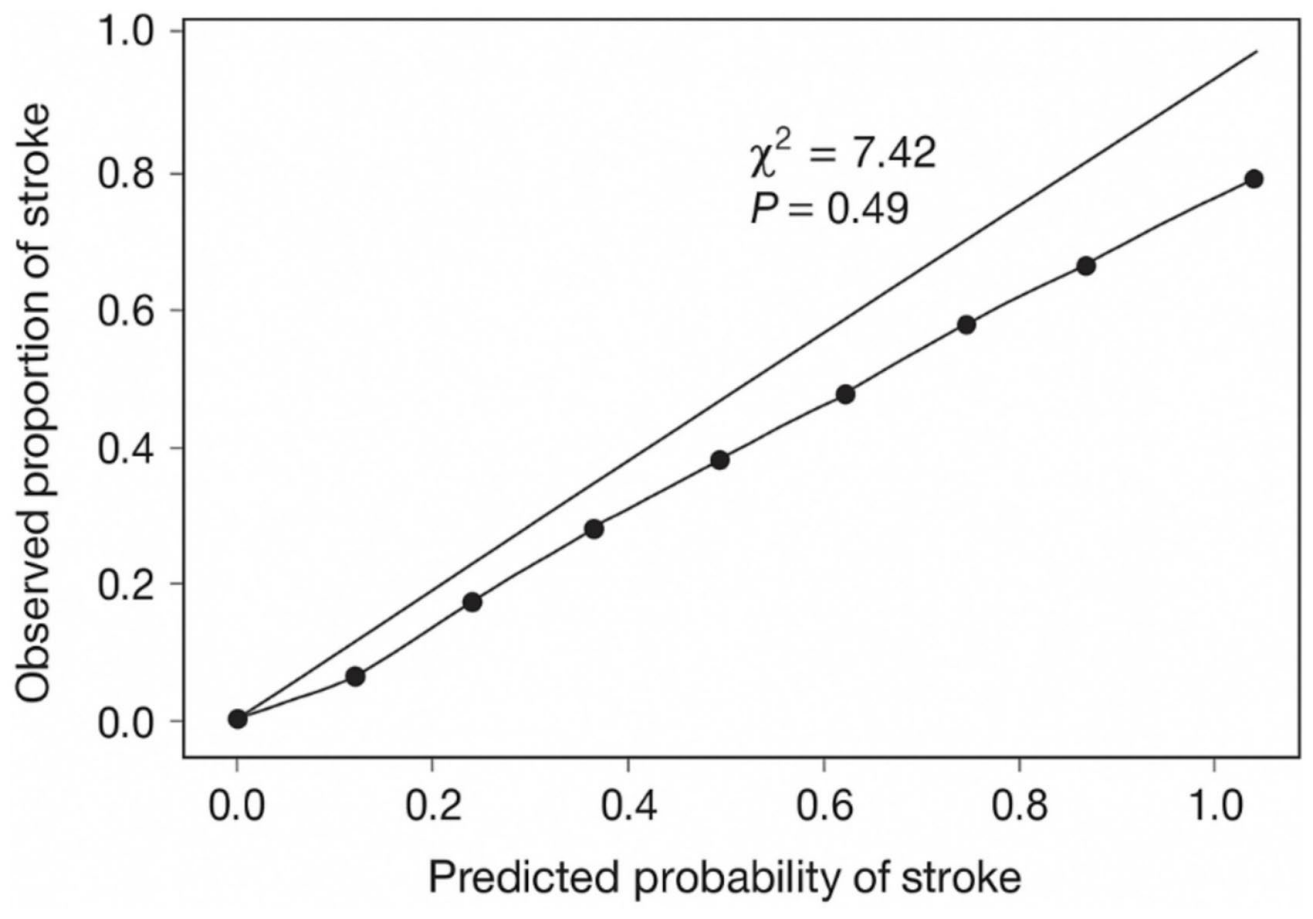
Hosmer-Lemeshow calibration plot for the multivariable logistic regression.

Multivariate logistic regression analysis showed that several plaque characteristics, including moderate to high echogenicity, bifurcation location, plaque length >10 mm, intraplaque hemorrhage, higher neovascularization grade, and lower proportion of hard regions, were independently associated with increased stroke risk as shown in Table 5. The model achieved an AUC of 0.81 (95% CI: 0.74–0.88), indicating good discriminatory performance. Model calibration was evaluated using the Hosmer–Lemeshow goodness-of-fit test (χ^2^ = 7.42, *p* = 0.49), confirming adequate fit as shown in Figure 6. IMT was not independently significant and was therefore excluded from the final model. Full multivariable logistic regression outputs, including coefficients, standard errors, odds ratios, 95% confidence intervals, and *p*-values, are provided in Table 5, and the Hosmer-Lemeshow plot is included as show in Figure 6.

**Table 5:** Multivariate logistic regression analysis of independent ultrasound plaque predictors of stroke risk.

Index	Coefficient	Std error	OR (95% CI)	*p*
Echogenicity distribution (Low/Medium-High)	−0.86	0.35	0.43 (0.22, 0.84)	0.01
Location distribution (Carotid bifurcation/Other)	0.67	0.32	1.95 (1.03, 3.66)	0.04
Plaque length >10 mm	0.79	0.33	2.20 (1.15, 4.22)	0.02
Intraplaque hemorrhage	0.76	0.34	2.13 (1.09, 4.17)	0.03
Calcification degree (None/Mild vs. Moderate/Severe)	−0.46	0.34	0.63 (0.33, 1.22)	0.17
IMT > 1 mm	0.64	0.34	1.90 (0.98, 3.68)	0.06
Neovascularization grade (0/1 vs. 2/3)	0.86	0.35	2.35 (1.19, 4.64)	0.01
Proportion of hard regions	−0.12	0.05	0.89 (0.81, 0.97)	0.01

## 4 Discussion

This retrospective study explored the relation between the ultrasound features of carotid plaques and stroke risk and showed that different ultrasound features are predictors of stroke risk within the upcoming year. This ground-breaking work enhances clinical knowledge of the cerebrovascular risk for the first time looking at the specific features of the plaques, rather than their general classification as unstable or other plaques, and this way elucidates even more the biology and the mechanisms and the physiopathology cerebrovascular disease. This is because, while plaque echogenicity is a marker of tissue microarchitecture within a plaque, the association demonstrated with stroke risk is that plaque echogenicity is inversely associated with stroke risk. When echogenicity is low, the plaque is a hemorrhagic one; when it is medium or high, it is a fibrous one. Moreover, there is a notable risk of intracranial hemorrhage associated with soft plaques, and the plaque microarchitecture is one that is rich in hemorrhagic tissue (31, 32).

The findings of the study confirm that plaques containing medium to high levels of echogenicity correspond to lower risk of stroke, substantiating that ultrasounds can differentiate between stable and unstable plaques. In contrast, plaques with low levels of echogenicity correspond to a higher risk due to the possibility of the plaques being soft and lipid-dense, associated with inflammatory cellular infiltration, rupture, and immature neovascularization, regions that are prone to rupture (33). These high echogenicity plaques contain a lower risk of rupture due to their structural integrity and because they contain collagen-rich or calcified matrices that reduce embolic potential (34). These findings also confirm that ultrasounds have the clinical potential to detect the presence of atheroclastic plaques within the carotids and to assess a risk potential of certain plaques and tissue via the tissue composition of the plaques (35).

Risk of stroke can be assessed based only on the presence of plaques, particularly on the carotid bifurcation. The bifurcation’s complex hemodynamics consisting of disrupted laminar flow and low shear stress accelerates the atherosclerotic process and the development of hazardous atherosclerotic plaques (36). The abnormal flow dynamics are also the primary cause of the bifurcation’s tendency of forming plaques of such significant risk (37). The disrupted laminar flow, low shear stress, and flow reversal present in the carotid bifurcation create mechanical strain on the endothelium which is associated with the activation of atherosclerosis-promoting biochemical cascades. The hemodynamics dictate plaques that are perilous in every respect from progressive enlargement, to surface erosion, to complete rupture. Plaques at arterial bifurcations also contribute to stroke risk by acting as embolic reservoirs and hemodynamically significant stenoses. The diseased plaques that form at the arterial bifurcations heighten the risk of stroke by hemodynamically significant stenoses of the arterial lumen (38). The plaques discovered at the bifurcations of the arteries are known to elevate stroke risk by narrowing the artery’s lumen (39).

Stroke incidence had significant associations with intraplaque hemorrhage. The rupture of plaque’s weak neovessels releases blood, causing oxidative stress and inflammation that weakens the cap fibroplasia (40). The fragile neovessels that are usually formed by hypoxia-driven angiogenesis, are present in the atherosclerotic core. The ruptured immature neovessels located within the plaque are able to release blood (41). The oxidative stress, inflammation, and the neutrophils that are present within the plaque, are a result of de-oxygenated blood and its byproducts (28). Fibrous cap weakening due to these processes, rupturations, and ensuing cerebral events (42). These findings highlight the importance of detecting intraplaque hemorrhage, as documented in the studies that utilized ultrasound, MRI, and histopathology, for plaque instability (43, 44).

Patients who had a stroke later had higher levels of neovascularization within their plaque, measured with contrast-enhanced ultrasound. The presence of microvessels in a plaque has become regarded as a key sign of ruptured plaque (45). The vascularization of a plaque is caused by the presence of hypoxic tissues within the thickened intima of the artery in combination with excess production of proangiogenic factors such as vascular endothelial growth factor (VEGF) (46). While the growth of additional blood vessels may be expected, newly formed vessels, on their own, tend to be poorly constructed. Lacking one of the pivotal structural elements, pericytes, and their outer matrix, the vessels become particularly prone to structural failure. Thus, they tend to become increasingly permeable, allowing intra-plaque hemorrhage and the entry of inflammatory factors and cells into the plaque (47). Therefore, because they are more prone to hemorrhage and tend to rupture, plaques with a higher vascularization are at a greater risk of forming a thrombosis (48).

Plaque that measures longer than 10 mm with an Atherogenic burden and vulnerable tissues such as bleeding tissues and neovessels, together, increases the odds for increased plaque burden (49). Furthermore, large plaques comprise neovascular tissues and hemorrhagic volumes, both of which underpin and add to the underlying risk (50). These examples underscore the importance of the shape of atherosclerosis instead of just the presence of such an ailment. It was noted that, as evidenced by a smaller proportion of fibrous regions and the computed Young’s moduli (which of the elastography soft tissues), decreased plaque consistency was linked with increased cerebrovascular accident risk, especially since the mean Young’s moduli between groups was statistically uniform. While mean plaque stiffness was similar among groups, this strongly suggests that the texture of plaque (the presence of softer, thus more mobile regions within a plaque), as opposed to generalized plaque stiffness, was more reflective of its susceptibility to that plaque. Regions that are lipid-laden, and comprise soft necrotic tissues that are subject to Shear planes, are at a higher risk of fracture. Plaques that are rigid exhibit a pattern of fibrous and calcified tissues, which results in uniform stiffness and detracts from plaques that are symptomatic (51). Although increased IMT was recorded in stroke patients, it did not remain independently significant after adjustments for treatment multivariate, implying that features related to plaques are more predictive than generalized artery thickening.

The implications of the results here are important, especially in relation to clinical management of patients with asymptomatic carotid artery diseases. This research illustrates the additional prognostic value that can be derived from the evaluation of individual plaque characteristics, as revealed by ultrasound. These findings are undoubtedly clinically actionable. Currently, guidelines place primary importance on the percentage of stenosis as the sole basis upon which clinical decisions are made for intervention. This, however, should not be the case, as our study findings point out the other plaques, as well as their microstructural features, which possess substantial prognostic value and should be analyzed in addition to the significant stenosis. Enhanced ultrasound, such as the contrast enhanced ultrasound and elastography, can provide the means to identify high-risk patients. This, in turn, may reduce the number of patients who are at low risk and prevent unnecessary interventions in the low-risk patients.

In addition, the implications of our study have the potential to refine artificial intelligence (AI) and deep learning techniques for the automated recognition and assessment of carotid plaques, significantly enhancing reproducibility and scalability in clinical processes, particularly in risk assessment and stratification. Recent studies have demonstrated that deep learning models can markedly enhance the clinical potential of carotid plaque detection, improving diagnostic accuracy, sensitivity, and reducing clinician workload and diagnostic time (52). By integrating the findings that underscore the prognostic value of individual plaque characteristics beyond the degree of stenosis, AI algorithms can be trained to recognize and evaluate a broader range of ultrasound features and microstructural attributes (53). This approach allows for the development of sophisticated models that can analyze complex imaging data, leading to more accurate identification of high-risk patients. As these AI systems learn from diverse datasets that include various plaque features they can improve their predictive capabilities, ensuring that clinicians receive reliable assessments that inform treatment decisions.

Nevertheless, it is important to acknowledge the limitations of this study. First, as a retrospective cohort study conducted at a single tertiary hospital, the findings may be subject to selection bias and may not be generalizable to broader populations or other healthcare settings. Second, the reliance on operator-dependent ultrasound imaging introduces potential variability in plaque characterization, even though standardized protocols were followed. Multicenter studies with consistent imaging protocols are needed to validate these findings. Third, the one-year follow-up period may not capture the long-term stroke risk associated with carotid plaque features. Extending follow-up durations could provide deeper insights into the temporal relationships between plaque instability and cerebrovascular events. In addition, although multivariate regression was employed to control for confounding factors, residual confounding cannot be entirely excluded. The study also did not incorporate advanced imaging modalities such as magnetic resonance imaging or computed tomography angiography, which could complement ultrasound findings by providing additional structural and compositional details. Future research should focus on prospective, multicenter studies with larger and more diverse cohorts to confirm the predictive value of ultrasound-derived plaque features. Integration of multimodal imaging approaches and machine learning models may enhance the precision of risk stratification. Furthermore, exploring the impact of targeted interventions based on plaque phenotype could provide actionable insights for personalized stroke prevention strategies.”

## 5 Conclusion

This study demonstrates that specific ultrasound imaging features of carotid plaques (echogenicity, anatomical location, plaque length, intraplaque hemorrhage, neovascularization grade, and plaque hardness) are independently associated with stroke risk. These findings highlight the importance of evaluating plaque composition and stability alongside luminal stenosis for ischemic stroke risk stratification. Advanced ultrasound modalities, including contrast-enhanced imaging and elastography, provide effective tools for identifying high-risk patients who may benefit from targeted preventive interventions. Despite its limitations, this study provides a basis for integrating imaging biomarkers into clinical practice and underscores the need for further research to validate and extend these observations. By shifting the emphasis from stenosis severity to plaque vulnerability, this approach has the potential to improve stroke prevention and patient outcomes.

## Data Availability

The original contributions presented in the study are included in the article/supplementary material, further inquiries can be directed to the corresponding author/s.
